# Estimated annual prevalence, medical service utilization and direct costs of lung cancer in urban China

**DOI:** 10.1002/cam4.3845

**Published:** 2021-03-21

**Authors:** Dawei Zhu, Xuefeng Shi, Stephen Nicholas, Yong Ma, Ping He

**Affiliations:** ^1^ China Center for Health Development Studies Peking University Beijing China; ^2^ School of Management Beijing University of Chinese Medicine Beijing China; ^3^ National Institute of Chinese Medicine Development and Strategy University of Chinese Medicine Beijing China; ^4^ Research Institute for International Strategies Guangdong University of Foreign Studies Guangzhou China; ^5^ School of Economics and School of Management Tianjin Normal University Tianjin China; ^6^ TOP Education Institute Sydney NSW Australia; ^7^ Newcastle Business School University of Newcastle Newcastle NSW Australia; ^8^ China Health Insurance Research Association Beijing China

**Keywords:** annual prevalence, China, lung cancer, medical service utilization, treatment costs

## Abstract

**Background:**

Evidence‐based policy making for delivering affordable lung cancer care relies on the breadth, depth and quality of knowledge of its treatment costs. This study estimates the annual prevalence, medical service utilization and direct treatment costs of lung cancer in urban China.

**Materials and Methods:**

Using claim data from China's urban basic medical insurance between 2013 and 2016, we constructed a nationally representative sample of lung cancer patients in urban China. Weighted descriptive analyses, Poisson regressions and generalized linear modelling were used to analyse lung cancer medical service utilization and costs and their associations with patient characteristics.

**Results:**

In urban China, the annual prevalence of lung cancer was 87.65/100000, with nearly 0.65% of total health expenditures of urban residents spent on lung cancer treatments. Weighted average annual total medical costs of lung cancer was RMB33.78 (US$5.36) thousand, with annual out‐of‐pocket costs of RMB10.26 (US$1.63) thousand. The average yearly number of lung cancer‐related outpatient visits was 2.42 and inpatient admissions was 2.07, with an average cost of RMB0.75 (US$0.12) thousand for outpatients and RMB 15.67 (US$2.49) thousand for inpatients. Inpatient expenses were the major component (95%) of lung cancer medical costs, with roughly 67% of inpatient services occurring in high‐level tertiary hospitals. Medical care utilization and direct medical costs were associated with sex, age and insurance status. Western medicine costs were the major contributor (39.4%) to average lung cancer‐related medical costs.

**Conclusion:**

Lung cancer imposed a significant economic burden on China's health system and a financial cost on lung cancer sufferers and their families. Specific policies are required to efficiently allocate health resources, contain health expenditure and decrease the individual financial burden of lung cancer.

## Graphical abstract

In urban China, the annual prevalence of lung cancer was 87.65/100000, with nearly 0.65% of total health expenditures of urban residents spent on lung cancer treatments. Inpatient expenses were the major component (95%) of lung cancer medical costs.Western medicine cost contributes 39.4% for lung cancer medical costs.

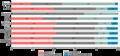

## 1 INTRODUCTION

Lung cancer is the leading cause of cancer incidence and cancer mortality globally,^1^ imposing substantial financial burdens on individuals, families and a country's health system. In 2018, it was estimated that there was 2.09 million new lung cancer cases and 1.76 million deaths, accounting for 18.4% of all global cancer deaths.^2^ Lung cancer treatment costs accounted for between 15% and 20% of the total cancer treatment costs in the United States and European countries,^3^, ^4^ and 4% of total healthcare cost in Iran.^5^ With the emergence of new techniques and treatment options for lung cancer patients, the lung cancer economic burden on families and the health system will continue to rise.^6^

China faces severe challenges in meeting its lung cancer burden. In China, lung cancer has been the most common cancer and the first leading cause of cancer death for several years.^7^, ^8^ China contributed to 37% of all new lung cancer cases and 39% of lung cancer deaths globally, while accounting for only 19% of the world's population.^2^, ^9^ Compared to global lung cancer incident rates, China's rate is high, with a steadily increasing crude incidence over the past decade,^7^, ^10^ and the lung cancer mortality rate is also high compared to other countries.^11^ As the most populous country in the world, with a rapidly ageing population and high incident and mortality rates, the burden of lung cancer will significantly increase in China.

The economic burden caused by lung cancer is not well researched in China. Sporadic studies on the economic burden of lung cancer in China were carried out for specific regions, such as Beijing and Guangxi, or based on data with poor representativeness, comprising only a few hospitals, making it difficult to draw nationwide conclusions.^9^, ^12^, ^13^, ^14^ The estimated average cost per lung cancer inpatient admission ranged from US$2220 to US$4612, with the estimated mean medical expenditure within 1 year after lung cancer diagnosis US$9788.^9^, ^13^, ^14^ Using data from secondary, tertiary general and specialized cancer hospitals, Cai et al estimated the direct medical costs of lung cancer treatments in China of RMB24.3 billion.^15^ But, the exclusion of primary healthcare facilities meant that the economic burden of lung cancer was underestimated. In addition, Cai et al did not analyse the economic burden on individuals, or did they provide detailed treatment costs stratified by age, gender and type of health insurance.

With the rapid increase in China's aging population, the emergence of new techniques and treatment options and increased survival rates, accurate estimates of lung cancer treatment cost are crucial for health policy making. Understanding the demographic characteristics of lung cancer patients and their patterns of medical services use is needed to rationalize the allocation of health resources for lung cancer treatment and prevention and to assess the cost‐effectiveness of specific prevention and treatment lung cancer interventions. We accessed China's Urban Employee's Basic Medical Insurance (UEBMI) and Urban Resident's Basic Medical Insurance (URBMI) claims data, which provided sociodemographic information and medical cost data on more than 95% of all urban residents in China between 2013 and 2016.^16^ Using the unique data, our study estimates the annual prevalence and total direct medical expenditure on lung cancer treatment in urban China; assesses medical care utilization measured by outpatient visits and inpatient admissions and investigates the distribution of medical costs across different lung cancer treatments.

## 2 MATERIALS AND METHODS

### 2.1 Data source

In the 2013–2016 period, UEBMI, for the urban employed, and URBMI, for urban unemployed, children and students, were the two main social health insurance schemes in urban China which covered 95% of all urban residents in mainland China.^16^ Using stratified random sampling method, sample cities were selected, and 5% random sample of UEBMI and URBMI beneficiaries’ insurance claims in sample cities collected by the China Health Insurance Research Association (CHIRA)^17^ in 2013–2016. This cross‐sectional data contain lung cancer beneficiaries’ demographic information, insurance type, diagnoses, inpatient–outpatient services and detailed medical costs. The CHIRA sampling design assigned a sample weight to each beneficiary and the use of these weights permitted calculations of nationally representative lung cancer medical service utilization rates and treatment costs. The total population count of UEBMI and URBMNI insured members stratified by age and gender was obtained from China's Labor Statistical Yearbook and the annual national sample survey on population changes.^18^

### 2.2 Sample and Measures

From the CHIRA database, we used the International Classification of Diseases, 10th version (ICD 10) codes (I34.0‐I34.9 as the primary diagnosis),^19^ to identify 38199 lung cancer patients with 114992 lung cancer‐related medical records between 2013 and 2016. Medical care utilization included the number of annual outpatient visits, comprising pharmacy, primary care, secondary and tertiary hospitals and the number of annual inpatient admissions to primary care facilities, secondary hospitals and tertiary hospitals. In China, healthcare is delivered via a three‐tiered system, comprising primary care facilities, including village clinics, township hospitals and community health centres; secondary county and district hospitals with 100–499 beds, providing comprehensive health services, medical training and regional‐based research; and tertiary municipal hospitals with over 500 beds, providing complex healthcare, medical education and research.^20^ Direct medical costs, including spending on western medicine, traditional Chinese medicine (TCM), medical services and diagnostic tests/medical consumables, were measured by the average cost per visit and average out‐of‐pocket (OOP) cost per visit. OOP spending was the direct cash payment not reimbursed by health insurance. Control variables included age groups (younger than 45, 45–54 , 55–64, 65–74 and 75 years or older), gender (male and female), insurance type (UEBMI and URBMI) and year (2013, 2014, 2015 and 2016).

### 2.3 Statistical analysis

To estimate the prevalence of lung cancer, the numerator was the total number of beneficiaries in the database who met our definition of lung cancer for the total sample and each of the basic demographic characteristics and the denominator was the total population count of UEBMI and URBMI insured. Weighted descriptive analyses were used to analyse the sample characteristics, medical service utilization and medical costs. Associations between medical service utilization and sociodemographic characteristics were evaluated by Poisson regression. A generalized linear model (GLM) with a gamma distribution and a log link was used to assess the association of average total costs and OOP cost with patient sociodemographic characteristics. Since our primary interest was to assess lung cancer‐related medical service utilization and direct medical costs, the coefficients were transformed back to the original scale. To calculate annual visits or cost for any patient, the addition factors for each of the characteristics for that patient were added onto the baseline female, age less than 45, with URBMI in 2013 estimate.

Unless otherwise specified, all descriptive statistics and GLM estimates in the text and tables are weighted to have national estimates based on the sample weight provided by CHIRA. A p‐value of less than 0.05 was considered statistically significant. The software Stata version 15 for Windows (Stata Corp, College Station, TX, USA) was used for the statistical analysis.

## 3 RESULTS

### 3.1 Sample characteristics

The first three columns of Table 1 display the original and weighted sample characteristics. There are 38.2 thousand patients with lung cancer in our sample, and the weighted number was 2256.21 thousand. Among all lung cancer patients, 62.96% were men, 51.47% were aged 65 years or older and 70.67% were covered by UEMBI.

**TABLE 1:** Characteristics of sample, prevalence and direct medical expenditure of lung cancer, 2013 to 2016.

	Sample, Thousands (%)	Weighted sample, Thousands (%)	Annual prevalence, 1/100000 (95% CI)	Total Direct medical expenses Billion RMB (95% CI)	Annual per person Direct medical expenses Thousand RMB (95% CI)	Annual OOP per person expenses Thousand RMB (95% CI)
Gender						
Female	13.45 (35.20)	836.92 (37.04)	66.37 (66.22,66.51)	24.94 (22.72,27.16)	29.81 (27.16,32.46)	9.86 (8.88,10.83)
Male	24.75 (64.80)	1422.28 (62.96)	108.04 (107.86,108.22)	51.36 (47.52,55.20)	36.12 (33.42,38.82)	10.50 (9.90,11.11)
Age group						
0–44	1.87 (4.89)	92.35 (4.09)	5.49 (5.45,5.53)	2.94 (2.51,3.37)	32.16 (27.47,36.84)	11.99 (9.95,14.02)
45–54	5.07 (13.27)	331.20 (14.66)	81.55 (81.27,81.82)	10.41 (8.25,12.57)	31.37 (24.86,37.89)	11.40 (8.66,14.14)
55–64	11.02 (28.85)	672.78 (29.78)	249.23 (248.64,249.83)	26.08 (22.84,29.32)	38.79 (33.97,43.61)	12.44 (11.36,13.51)
65–74	10.89 (28.51)	567.59 (25.12)	399.21 (398.17,400.25)	19.19 (18.18,20.21)	33.79 (32.00,35.58)	9.89 (9.23,10.56)
75+	9.35 (24.48)	595.28 (26.35)	656.11 (654.45,657.77)	17.68 (14.89,20.47)	29.70 (25.02,34.39)	7.26 (6.66,7.87)
Insurance						
URBMI	9.96 (26.07)	662.64 (29.33)	46.13 (46.02,46.25)	18.99 (17.61,20.38)	28.66 (26.57,30.75)	13.09 (12.04,14.14)
UEBMI	28.24 (73.93)	1596.57 (70.67)	139.85 (139.63,140.07)	57.31 (52.80,61.83)	35.90 (33.08,38.73)	9.09 (8.46,9.72)
Total	38.20 (100.00)	2259.21 (100.00)	87.65 (87.54,87.77)	76.31 (71.57,81.05)	33.78 (31.68,35.88)	10.26 (9.72,10.80)
Total Health Expenditure of Urban Residents (2013 to 2016), Billion RMB						11697.7
Total direct medical expenses as per cent of total Health Expenditure of Urban Residents, %						0.65
Average annual direct medical expenses as per cent of GDP per capita, %						69.56
Average annual OOP cost as per cent of non‐food household expenditure among urban residents, %						70.47

UEBMI, Urban Employee's Basic Medical Insurance; URBMI, Urban Resident's Basic Medical Insurance; OOP, out‐of‐pocket.

### 3.2 Annual prevalence and direct medical expenditure

As shown in the fourth column of Table 1, the 2013–2016 annual prevalence of lung cancer was 87.65 cases per 100,000 people. The annual prevalence was higher among men (108.04 per 100000) than women (66.37 per 100,000); and those aged 75 or older had the highest prevalence (656.11 per 100,000).

The estimated total direct medical cost for lung cancer treatment in urban China was RMB76.31 billion (US$12.11 billion) during 2013–2016, or roughly RMB19.08 billion (US$3.03 billion) per year. The cost for male patients was twice that for female patients and patients aged 55–64 years had the highest total direct medical expenses. The weighted average annual direct medical costs per lung cancer patient was RMB33.78 thousand (US$5.36 thousand), with 30.37%, or RMB10.26 thousand (US$1.63 thousand), OOP expenses. The proportion of health expenditure spent on lung cancer in the total health expenditure of urban residents was 0.65% on average during 2013 to 2016. The average annual direct medical expenses per patient accounted for 69.56% of per capita GDP, and the average annual OOP spending accounted for 70.47% of average non‐food household expenditure.

### 3.3 Annual utilization of medical services and associated costs

Table 2 shows outpatient visits and inpatient admissions as a measure of medical service utilization, with their associated costs. The annual number of outpatient visits per lung cancer patient was 2.42, incurring, on average, RMB745.64 (US$118.36) per visit with 33.15% of the total costs OOP expenses. The annual number of inpatients admissions per lung cancer patient was 2.07, incurring an average cost of RMB15669.53 (US$2487.23) per inpatient with RMB 4744.43 (US$753.08) OOP expenses. Inpatient costs accounted for 96% of annual cost of lung cancer treatments, and roughly 67% of inpatient services occurred in tertiary hospitals. Tertiary hospitals had higher average inpatient cost (RMB17452.70; US$2770.27) than secondary hospitals (RMB12893.51; US$2046.59) and primary care facilities (RMB8454.78; US$1342.03). OOP cost displayed a similar pattern to total costs by healthcare facility.

**TABLE 2:** Summary of annual utilization of medical care and associated costs, weighted mean (SD).

	Average annual visit number	Average cost per visit, RMB	Average out‐of‐pocket cost per visit, RMB
Outpatient visit (Total)	2.42 (7.16)	745.64 (1851.65)	247.18 (1063.70)
Pharmacies	0.04 (0.57)	559.52 (1025.10)	398.20 (559.44)
Primary care	0.51 (3.65)	848.59 (1728.99)	96.86 (405.61)
Secondary hospitals	1.56 (5.08)	446.76 (1239.17)	157.25 (379.78)
Tertiary hospitals	0.31 (2.06)	775.94 (1995.96)	310.52 (1284.70)
Inpatient admission (Total)	2.07 (2.54)	15669.53 (18729.38)	4744.43 (8905.44)
Primary care facilities	0.13 (1.03)	8454.78 (14520.81)	2205.10 (7342.99)
Secondary hospitals	0.56 (1.24)	12893.51 (14508.19)	3656.09 (5042.18)
Tertiary hospitals	1.39 (2.30)	17452.70 (20231.15)	5417.07 (10095.03)

### 3.4 Predictors of medical utilization and costs

Table 3 shows the association between medical utilization and medical costs with patient sociodemographic characteristics. The baseline represents utilization and medical costs for an under 45‐year‐old woman with URBMI in 2013. When a patient displays any of the characteristics listed in Table 3, the annual utilization and medical costs are estimated by the sum of the baseline and the addition costs corresponding to each patient's characteristics. For example, the annual number of outpatient visits for an under 45‐year‐old woman with URBMI in 2013 was 0.798, and for a man was 0.398 (0.798–0.400), and the associated medical cost for an under 45‐year‐old woman with URBMI in 2013 was 1510.412 and 1523.867 (1510.412 + 13.455) for a male outpatient.

**TABLE 3:** Utilization and medical cost associated with sociodemographic characteristics and year.

	Outpatient			Inpatient		
	The average number of annual visits	Average cost per visit (RMB)	Average OOP cost per visit (RMB)	The average number of annual admissions	Average cost per admission (RMB)	Average OOP cost per admission (RMB)
**Baseline**	0.798 (0.793,0.803)	1510.412 (1489.143,1531.681)	782.002 (762.096,801.909)	1.390 (1.382,1.397)	15193.700 (15096.787,15290.613)	8162.681 (8081.837,8243.526)
**Increment**						
Gender						
Male	−0.400 (−0.403,−0.396)	13.455 (9.908,17.003)	−45.668 (−47.881,−43.456)	0.276 (0.272,0.279)	588.830 (553.189,624.471)	10.918 (−6.008,27.843)
Age groups						
45–54	−0.126 (−0.135,−0.117)	33.187 (22.019,44.355)	−0.142 (−8.967,8.683)	0.127 (0.117,0.138)	−1344.685 (−1447.720,−1241.649)	−670.564 (−728.049,−613.079)
55–64	0.175 (0.166,0.184)	−104.986 (−115.124,−94.847)	−147.492 (−155.329,−139.655)	0.623 (0.613,0.633)	−1830.156 (−1927.334,−1732.979)	−1217.065 (−1271.192,−1162.939)
65–74	0.325 (0.316,0.334)	−121.455 (−131.622,−111.288)	−145.740 (−153.581,−137.898)	0.134 (0.124,0.144)	−888.915 (−988.295,−789.535)	−1555.022 (−1609.516,−1500.528)
75+	−0.282 (−0.291,−0.274)	−163.833 (−174.058,−153.609)	−137.670 (−145.599,−129.741)	−0.234 (−0.243,−0.224)	307.900 (206.330,409.471)	−1830.205 (−1884.755,−1775.655)
Insurance Type						
UEBMI	0.736 (0.732,0.739)	80.045 (76.090,84.000)	6.850 (4.351,9.349)	0.107 (0.103,0.111)	2708.850 (2673.794,2743.905)	−2097.292 (−2118.566,−2076.019)
Year						
2014.	−0.070 (−0.073,−0.066)	−716.375 (−727.329,−705.420)	−197.153 (−204.003,−190.302)	0.123 (0.118,0.128)	−1595.243 (−1650.388,−1540.098)	−157.350 (−182.781,−131.920)
2015.	1.315 (1.310,1.320)	−718.489 (−729.059,−707.920)	−311.237 (−317.525,−304.949)	0.496 (0.490,0.501)	−113.239 (−169.892,−56.587)	7.776 (−17.898,33.449)
2016.	1.468 (1.462,1.473)	−972.106 (−982.418,−961.795)	−266.716 (−273.060,−260.372)	0.768 (0.763,0.774)	−1920.624 (−1975.092,−1866.156)	−306.583 (−331.641,−281.524)

The baseline represents the utilization and cost for an under 45‐year‐old woman with URBMI in 2013.

UEBMI, Urban Employee's Basic Medical Insurance; URBMI, Urban Resident's Basic Medical Insurance; OOP, out‐of‐pocket.

From Table 3, men utilized less outpatient services, but more inpatient services with higher average cost per visit, than women. Patients aged 45–74 years old used more inpatient services, with a low average cost per admission. The total inpatient cost per visit was higher for patients covered by UEBMI than patients covered by URBMI, while the OOP cost was higher for URBMI patients than UEBMI patients. Compared with 2013, the average number of inpatient admissions increased over the 2014–2016 period, while average costs decreased.

### 3.5 Structure of lung cancer medical costs

**FIGURE 1:**
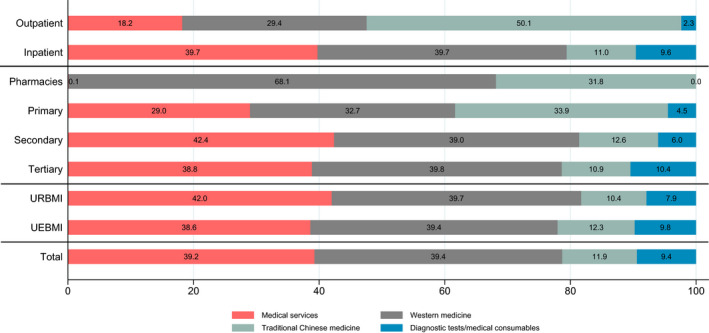
Cost subtypes distribution of patients with lung cancer (percentages).

The distributions of various medical costs are presented in Figure 1. The highest cost was western medicine (39.4%), followed closely by medical services (39.2%). Diagnostic tests/medical consumables costs and TCM cost each accounted for about 10% of medical costs. TCM accounted for substantial proportion of medical costs in lower level health facilities and outpatient services; high‐level hospitals had a high proportion of diagnostic tests/medical consumables costs.

## 4 DISCUSSION

Using urban basic medical insurance claims data, our study provides a comprehensive estimate of the annual prevalence, medical service utilization and direct medical costs of lung cancer patients in urban China. With a nationally representative sample of more than 38 thousand lung cancer patients, we found the annual prevalence of lung cancer was 87.65 cases per 100000 people; nearly 0.65% of China's total health expenditures by urban residents was spent on lung cancer treatments; and the average annual direct medical costs of lung cancer patients was RMB33.78 thousand (US$5.36 thousand), with out‐of‐pocket costs of RMB10.26 thousand (US$1.63 thousand) or 30.37% of total lung cancer costs. The average number of lung cancer‐related outpatient visits was 2.42 and inpatient admissions was 2.07, with an average cost of RMB0.75 thousand (US$0.12 thousand) per outpatient and RMB15.67 thousand (US$2.49 thousand) per inpatient admission. Inpatient costs were the major component (95%) of total lung cancer medical costs. Lung cancer‐related medical care utilization and direct costs were associated with gender, age and insurance status. Medication expenses, especially western drugs, were the major contributor to average lung cancer‐related medical costs.

Our annual prevalence of lung cancer in urban China, 87.65/100000, was slightly higher than Zheng et al. 74.9/100,000 estimate using 2011 cancer registry data.^21^ This is likely because of the increase in the crude incidence and 5‐year relative survival rate in recent years.^1^, ^22^ Also, compared to 2011, the men–women ratio of prevalence decreased slightly, from 1.86 to 1.63 in our study. This is consistent with China's cancer statistics that show the incidence gap between males and females narrowed in this period.^1^, ^7^, ^23^

Nearly 0.65% of total health expenditures of urban residents were spent on lung cancer medical costs, which was similar to the European Union.^4^ We estimated the annual treatment cost of lung cancer in urban China as roughly RMB19 billion (US$3 billion). This was about 86% of national healthcare expense on lung cancer (RMB22 billion (US$3.5 billion) estimated by Cai et al.^15^ Our estimate of 86% for lung cancer treatment costs is also significantly higher than the total health expenditure of urban residents (77%) in national total health expenditures.^24^ The potential reason for our relative higher proportion of lung cancer in total lung cancer treatment was that Cai et al. did include primary healthcare facilities and the higher prevalence of lung cancer in urban compared to rural areas.^21^

The average annual cost per lung cancer patient and average cost per visit (outpatient and inpatient) in our study were lower than those estimated in some Chinese studies.^9^, ^25^ This may be due to the difference in locations, disease stages and demographic characteristics. Previous research has shown that higher‐level hospitals have higher cost than low ‐level hospitals^14^, ^26^ and first year cost of treatment was higher than subsequent years of treatment.^25^ While this is consistent with our data, additional studies should include more types of healthcare facilities and all stages of lung cancer patients.

Lung cancer imposes a substantial financial burden on individuals and families. Although the Chinese government has launched several policies to ease the financial burden of patients with lung cancer since 2006, such as price reductions in anticancer drugs^27^ and critical disease insurance,^15^, ^28^ OOP costs accounted for about 30% of total lung cancer expenses. Average OOP expenses were 70.47% of average non‐food household expenditure, which meant that a substantial proportion of families incurred catastrophic health expenditures, using the catastrophic threshold definition of 40% of non‐food household expenditure on health.^29^, ^30^ To mitigate this situation, a series of polices were initiated recently, including anticancer drug price negotiation, more anticancer drugs in drug list on social insurance and exempt tariffs on imported cancer drugs. We recommend further measures to protect mainly poor families from catastrophic lung cancer medical expenditures, such as adopting more anticancer drugs on the drug list and encouraging the use of generic medicines.

Consistent with previous studies in China, most of the lung cancer treatment costs were attributed to inpatient treatment,^31^ and the major inpatient service occurred in tertiary hospitals, with the highest medical cost per admission of all health facilities.^13^, ^14^, ^15^ One interpretation is that there was excessive health‐seeking behaviour by lung cancer patients for inpatient services provided by tertiary hospitals. The lack of ambulatory chemotherapy services^32^ and the low compensation level for outpatient lung cancer treatments are reasons for the high tertiary hospital admission rates.^33^ On the supply side, primary and secondary hospitals had a lower capacity to treat cancers than tertiary hospitals.^33^, ^34^ The evidence shows that there were less than 2 oncologists per 100,000 population in China, and oncologists were concentrated in high‐level tertiary hospitals.^34^ Unsurprisingly, patients bypassed lower‐level facilities to seek care in tertiary hospitals. In addition, the lack of a structured referral system permitted patients to choose their initial health facility for treatment, without referral by a lower‐level health facility to higher‐level hospitals, underscored this health‐seeking behavior.^35^

We found significant differences in medical service utilization and cost by gender, age and insurance type.^13^, ^14^ Compared with women, men had higher inpatient service utilization with higher treatment expenditures. A higher incidence of lung cancer in men suggests gender‐specific interventions for lung cancer, with different prioritization of cancer screening and treatment for males, leading to lung cancer treatment cost reductions. Due to the insurance schemes disparities in the types of medical services insured and their reimbursement rate, UEBMI patients had higher medical service utilization and higher total cost, but lower OOP expenses, than URBMI patients. UEBMI targeted urban employees who paid higher premiums, while URBMI targeted urban children, students, the unemployed and those with disabilities who paid lower premiums,^36^ which explains UEBMI's higher medical services and medications coverage and higher reimbursement rates. We found an increase trend in the number of clinical visits with a decrease trend in the cost per visit. The potential reason was the payment reforms in recent years in China, such as quota payment, where each inpatient episode was paid by a contracted quota, and diagnosis‐related group (DRG) payments, where hospitals receive a prospectively set fixed amount for each inpatient admission according to its DRG category. These policies encouraged hospitals to shorten average length of hospital stay and break one longer hospitalization into more shorter hospital admissions.^33^

Western medication was the leading cost component (39.4%) of lung cancer treatment costs, which is similar to other Chinese health studies,^13^, ^14^ but much higher than that in European Union (27%).^4^ According to Shi et al., chemotherapy was the most common treatment regimen among lung cancer patients in China, with more than 50% of patients receiving chemotherapy.^9^ Also, half of spending on anticancer medicines in China was for imported medicines, and anticancer drug prices in China are the second highest in the world.^37^ In contrast to higher‐level hospitals, TCM treatments accounted for substantial proportion of lower‐level health facilities services. Despite the lack of evidence on the clinical efficacy of TCM, TCM was widely used as supplementary drugs in cancer treatment in China.^28^, ^38^

This study has several policy implications. First, lung cancer causes huge economic pressure on China's health system, and it expected to increase with a rapid ageing population. This means the expansion of specific policies to control cancer creating behaviour, such as further taxes on tobacco consumption and better environmental pollution measures; to contain the cost of cancer treatment, including the early detection of lung cancer; to improve innovation ability of domestic drug companies; to reform provider payment mechanisms and to improve referral systems. Second, patients and their families bear a large financial burden when suffering lung cancer. The government should place more anticancer drugs on the drug list and encourage the use of generic medicines to reduce the OOP costs of anticancer drugs. Third, the government should design policies to provide training opportunities to oncologists in primary and secondary hospitals, and substantially improve their capacity, both to detect and treat lung cancer. Given the government's commitment to TCM, more high‐quality research should be supported to measure the effectiveness of TCM in the treatment of lung cancer.

This study has several limitations. First, our data do not allow us to estimate the cost stratified by pathological cancer subtypes and clinical stages of lung cancer. Second, our estimates do not consider treatment regimens given the lack of treatment regime information. Third, since the data we used were claim data, patients who did not go to medical care facilities or were not insured were not included in our sample. Fourth, the use of claim data may miss direct non‐medical cost and indirect cost of lung cancer patients, although this study focused on the direct medical cost. Previous studies with relatively small samples have shown that the direct non‐medical cost and indirect cost together account for 8.1% to 32.8% of total cost.^39^, ^40^ A national estimate of direct non‐medical cost and indirect cost should be addressed in future research. Fifth, there were some socioeconomic factors, such as education, income and the occupation, that were not controlled in our analysis. In addition, our data cannot allow us to distinguish the OOP cost into several categories, such as western medicine, medical services or TCM. Despite these limitations, our study used UEBMI and URBMI claims data that covered almost all urban Chinese and all types of medical care facilities, providing the first nationally comprehensive cost analysis for lung cancer patients in urban China.

## 5 CONCLUSION

Lung cancer imposes a significant economic burden on China's health system and a financial burden on cancer sufferers and their families. Our study provides a nation‐wide measure of the annual prevalence, direct medical expenditures and medical resource utilization by lung cancer sufferers in urban China. The greatest cost of lung cancer treatment was medication, especially western drugs. Already high by global standards, and with an ageing population, China's lung cancer incidence and survival rate will increase in the future, which will require more efficient allocation of health resources, improved healthcare services and the containment of treatment expenditures. Our reliable cost‐of‐illness data informs policy makers to implement cost control of western anticancer drugs and strengthen domestic drug companies; to develop more controls on treatment‐seeking behaviour; to improve early lung cancer detection; to reform the referral system and to expand the capacity of outpatient and lower‐level hospitals to upgrade their cancer treatment capacity. The Chinese government should adopt more anticancer drugs on the social health insurance drug list and encourage the use of generic medicines to decrease the medication financial burden on families. The efficacy of TCM lung cancer treatment needs urgent research to determine its effectiveness as an alternative to western drugs as a cancer treatment.

## 6 ETHICAL APPROVAL STATEMENT

Since the claims data were from an anonymized database, the Ethics Committee of Beijing University of Chinese Medicine deemed this study as exempt from ethical approval (No.2019BZHYLL0201). Since we used anonymized and de‐identified data, and no interventions were involved, written consent was neither required nor possible.

## CONFLICTS OF INTEREST

The authors declare no potential conflicts of interest.

## Data Availability

Data cannot be shared publicly because of secrecy agreement. Data are available from the China Health Insurance Research Association for researchers who meet the criteria for access to confidential data.
